# Cardiac Mechanics and Valvular and Vascular Abnormalities in Hypereosinophilic Syndrome

**DOI:** 10.3390/jcm13051403

**Published:** 2024-02-28

**Authors:** Attila Nemes

**Affiliations:** Department of Medicine, Albert Szent-Györgyi Medical School, University of Szeged, 6725 Szeged, Hungary; nemes.attila@med.u-szeged.hu

**Keywords:** hypereosinophilic, myocardial, mechanics, valvular, vascular, remodeling

## Abstract

Hypereosinophilic syndrome (HES) is considered to be a rare myeloproliferative disease that is characterized by persistent eosinophilia with associated multiple-organ damage. The heart is often involved in HES, representing a major cause of morbidity and mortality. HES is a heterogeneous group of disorders; the majority of the cases are idiopathic. Summarizing the findings regarding myocardial, valvular, and vascular abnormalities in a series of patients with HES, most studies found normal left ventricular (LV) volumes with reduced LV global longitudinal strain and LV apical rotation and twist in HES cases, accompanied by increased left atrial (LA) volumes and stroke volumes, reduced peak LA circumferential strain (representing systolic abnormalities), and mitral annular dilation and functional deterioration. Regarding the right heart, preserved right ventricular volumes and functional properties, increased right atrial volumes, mild RA functional abnormalities, and dilated tricuspid annular dimensions without functional impairment could be seen in these studies. Aortic and pulmonary valves showed no specific disease-related alterations. Vascular abnormalities included increased aortic stiffness without dilation of the aorta and pulmonary hypertension in some cases. These results suggest disease-specific but relatively mild myocardial, valvular, and vascular abnormalities in HES. The present review aimed to summarize the available clinical data about cardiac mechanics and valvular and vascular abnormalities in a series of patients with HES.

## 1. Hypereosinophilic Syndrome and the Heart

According to the international consensus group’s definitions, in the event of an absolute eosinophil count larger than 1.5 × 10^9^/L, with two measurements and/or the presence of tissue hypereosinophilia defined by a percentage of eosinophil cells in the bone marrow section exceeding 20% of all nucleated cells, and/or in the case where a pathologist is of the opinion that tissue infiltration by eosinophils is extensive and/or when marked deposition of eosinophil granule proteins is found (in the absence or presence of major tissue infiltration by eosinophils), hypereosinophilia is defined. The classification of hypereosinophilia is the following [1,2,3,4]:

− hereditary (familial clustering, pathogenesis unknown);
− primary (clonal, neoplastic, according to WHO criteria, underlying stem cell, myeloid or eosinophilic neoplasm, and eosinophils are considered neoplastic cells);
− secondary (reactive, non-clonal, including infections, asthma, and allergies, parasitic infestations, cytokine infusions, respiratory diseases, vasculitis, drug reactions non-hematological malignant diseases, connective tissue diseases, and non-Hodgkin’s and Hodgkin’s lymphomas);
− hypereosionophilia of an undetermined significance.

The first time the term hypereosinophilic syndrome (HES) was introduced was in 1968 by Hardy and Anderson, and the criteria for HES were determined in 1975 [5,6]. HES can be diagnosed if the HES criteria for peripheral blood hypereosinophilic are fulfilled, there is an organ damage/dysfunction attributable to tissue that is hypereosinophilic, and other disorders and conditions can be excluded in a background of organ damage. HES is a heterogeneous group of disorders, and the majority of HES cases are idiopathic [1,2,3,4]. 

Although almost all organs can be affected by hypereosinophilia-related damage, the most frequently affected organs are the heart, the skin, the nervous system, and the respiratory tract [7]. The heart is often involved in HES (54–73%), representing a major cause of mortality and morbidity in these cases [8,9]. Important long-known cardiac symptoms of HES are dyspnea, chest pain, cough, palpitations, embolic events, signs of congestive heart failure, murmur of mitral regurgitation, etc. [7,8]. A characteristic lesion seen in HES is eosinophilic myocarditis, which is a rare subtype of endomyocardial diseases. Cardiac damage caused by the infiltrating eosinophilic cells is known as “Loeffler’s endocarditis”, although, in the 1930s, Wilhelm Löffler demonstrated subjects with eosinophilic pneumonia caused by certain hookworms and parasites [10]. The clinical course of the disease can be divided into three stages:

(1) Necrotic (acute) stage: characterized by the infiltration and deposition of eosinophil cells, resulting in cytokine-mediated damage to the endocardium,
(2) Thrombotic stage: during which a layered thrombus is formed as a result of the activation of the tissue factor by the eosinophil cells accumulating in the myocardium;
(3) Fibrotic stage: the stage of myocardial fibrosis and its consequential wall stiffness.

In some circumstances, stage 2–3 cardiac HES is called Loeffler (or Löffler) endocarditis. HES is often classified as a restrictive cardiomyopathy. However, relatively little is known about HES-related cardiac mechanics and valvular and vascular abnormalities of this typical cardiomyopathy, characteristics which modern non-invasive imaging methods can help us determine [10]. It would also be important to summarize currently available findings regarding HES-associated cardiac abnormalities. 

## 2. Cardiovascular Imaging and MAGYAR-Path Study

During the last few years, cardiovascular imaging has undergone significant development. Computed tomography and magnetic resonance imaging (MRI) are now utilized in routine clinical practice, but major advancement has been seen in echocardiography as well. While decades ago, M-mode, two-dimensional, Doppler, and tissue Doppler echocardiography were part of the daily routine, completely new methods have appeared, like speckle-tracking echocardiography (STE) and volumetric three-dimensional (3D) echocardiography [11]. As a result, the detailed assessment of volumetric and functional properties of certain cardiac chambers and vascular functional evaluations have become available. Moreover, the complete assessment of myocardial mechanics together with functional evaluation of vasculature can be performed now using echocardiography. One of the latest developments is 3DSTE, a relatively new non-invasive echocardiographic tool which includes the advantages of the two new methods, enabling 3D visualization of the heart chambers, meaning that accurate volumetric measurements can be performed considering the heart cycle, with the evaluation of strains and the rotational parameters of cavities using the same acquired 3D echocardiographic datasets, using virtual 3D models [12,13,14,15]. The “Motion Analysis of the heart and Great vessels bY three-dimensionAl speckle-tRacking echocardiography in Pathological cases” (MAGYAR-Path) Study started in 2011 in the Cardiology Department of the University of Szeged, Szeged, Hungary. The aim of our study was to evaluate the clinical value of 3DSTE in certain disorders like HES. The results from the MAGYAR-Path study presented several abnormalities in the valvular and myocardial mechanics in HES [16,17,18,19,20,21].

The aim of the current review is to summarize findings about HES-related abnormalities in cardiac mechanics and vascular and valvular abnormalities in a series of patients with HES. The majority of the findings that will be discussed were first described in the MAGYAR-Path Study. Although most publications regarding HES are case reports, these have not been included in the present review, which discusses only papers involving series of patients with HES. From the vascular parameters presented, only those related to the aorta and the pulmonary artery have been mentioned.

## 3. The Left Heart and the Aorta

### 3.1. Left Ventricle

#### 3.1.1. Under Healthy Circumstances

**Figure 1:**
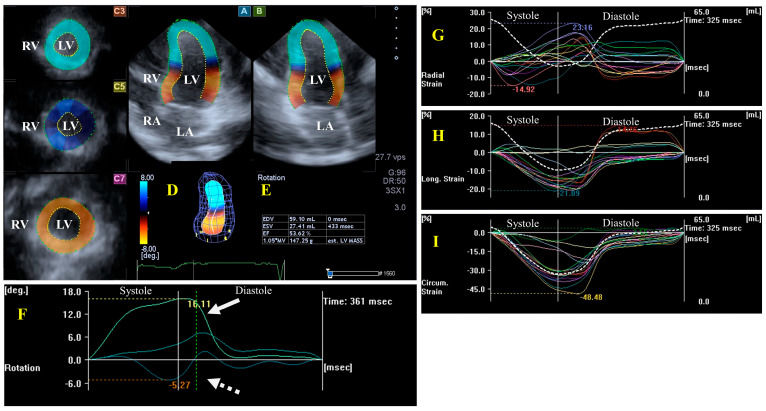
Evaluation of the left ventricle (LV) by three-dimensional (3D) speckle-tracking echocardiography. Typically, several views are automatically created during the LV assessment using a dedicated vendor-provided software: apical longitudinal four-chamber (**A**) and two-chamber (**B**) views and apical (**C3**), midventricular (**C5**), and basal (**C7**) short-axis views. In a 3D LV model (**D**), LV volumes, ejection fraction, and mass (**E**) and the apical (white arrow) and basal (white dashed arrow) rotations of the LV (**F**) are seen with time, as well as the LV global (white curve) and segmental (colored curves) radial (**G**), longitudinal (**H**) and circumferential (**I**) strain curves with time, and an LV volume changes curve with time (dashed white curve) are presented. Abbreviations: RA = right atrium; RV = right ventricle; MASS = LV muscle mass; LA = left atrium; LV = left ventricle; EF = ejection fraction; EDV = end-diastolic volume; and ESV = end-systolic volume.

The left ventricle (LV) is an egg- or bullet-shaped heart chamber located on the left side of the heart. Under healthy circumstances, during systole, the LV is emptied through the semilunar aortic valve (AV) towards the ascending aorta, with the mitral valve (MV) closed, while, during diastole, the LV fills up from the left atrium (LA) through the MV, with the AV closed. Without going into details about the exact structure of the LV, it is important to note that the LV has a special myocardial architecture: there are subepicardial fibers running in a left-handed direction, fibers in the mid-layer running circumferentially, and subendocardial fibers running in a right-handed direction. Subepicardial and subendocardial fibers are perpendicular to each other. The LV segments have a special 3D motion pattern having radial (thinning/thickening), longitudinal (lengthening/shortening), and circumferential (widening/narrowing) relaxation/contraction, which can be represented by echocardiographic unidimensional/unidirectional (LV-RS, LV-LS, and LV-CS, respectively) and combined multidimensional/multidirectional strains (LV area strain, LV-AS, and LV 3D strain, LV-3DS) [12,13,14,15,22]. The rotation of the LV basal regions is oriented clockwise during systole, while the rotation of the LV apical regions is counterclockwise, resulting in a motion called LV twist, a movement which is similar to wringing a towel [23,24]. During diastole, the basal and apical LV regions move in the opposite directions, resulting in a motion called LV untwisting. In some cases, LV twist is missing when the rotation of the basal and apical parts of the LV is the oriented in the same direction, clockwise or counterclockwise, a phenomenon which is named LV “rigid body rotation” (LV-RBR) [25] (Figure 1).

#### 3.1.2. In the Hypereosinophilic Syndrome

##### LV Structure, Volumes, and Hypertrophy

Although typical echocardiographic findings include endomyocardial thickening, no signs of LV wall thickening can be seen in most patients with HES [7]. Another typical echocardiographic finding is the fibrothrombotic obliteration of the ventricular apices [7]. According to the guidelines regarding the LV, typical criteria include endomyocardial plaques of a >2 mm thickness, tiny endomyocardial patches affecting more than one ventricular wall, a normal-sized ventricle, and enhanced M-movement of the interventricular septum [26,27]. Sixty-eight percent of patients with HES showed echocardiographic features of LV free wall thickening in another study [8]. Ventricular enlargement and decreased ventricular systolic function were found in a small group of patients with HES using echocardiography [28]. Cardiac MRI was used in patients with HES, and late gadolinium enhancement (LGE) imaging was found to be capable of detecting myocardial fibrosis and inflammation; in this study, LGE was more intense due to fibrosis than due to inflammation [29]. In another cardiac MRI study, regional LV function and end-diastolic volume were normal in all patients with HES. Non-ischemic LGE were found in half of the population; LGE was distributed in all LV myocardial layers; and there was no evidence of regional wall motion abnormalities. One third of the patients in this study had more than two lesions. The maximum eosinophil counts were associated with LGE lesions. According to the Lake Louise criteria, one third of the patients demonstrated evidence of myocardial inflammation, and MRI evidence of myocarditis was associated with a higher blood eosinophil count [30]. 

##### LV Functional Properties, Strains, and Rotational Mechanics

In a 2DSTE study, global LV-CS and global LV-RS were similar in normal controls and in patients with HES, but the global LV-LS of the normal controls was significantly higher than that of the patients with HES, and a global LV-LS ≤ 17% was the best predictor of LV endocardial dysfunction [31]. Results from the MAGYAR-Path study, in a comparison with matched healthy subjects, no LV dilation but a reduced 3DSTE-derived global LV-LS could be detected in patients with HES, localized to the basal regions [16]. Moreover, reduced LV apical rotation and twist were present in patients with HES, being mostly in the early necrotic phase. LV-RBR was present only in two out of eleven patients with HES (18%), a ratio which is lower than that seen in other cardiomyopathy patients [17]. In a special case with advanced Loeffler syndrome, a significantly reduced and significantly prolonged LV apical rotation could be seen, suggesting “stuck” LV rotational mechanics [18].

### 3.2. Left Atrium

#### 3.2.1. Under Healthy Circumstances

**Figure 2:**
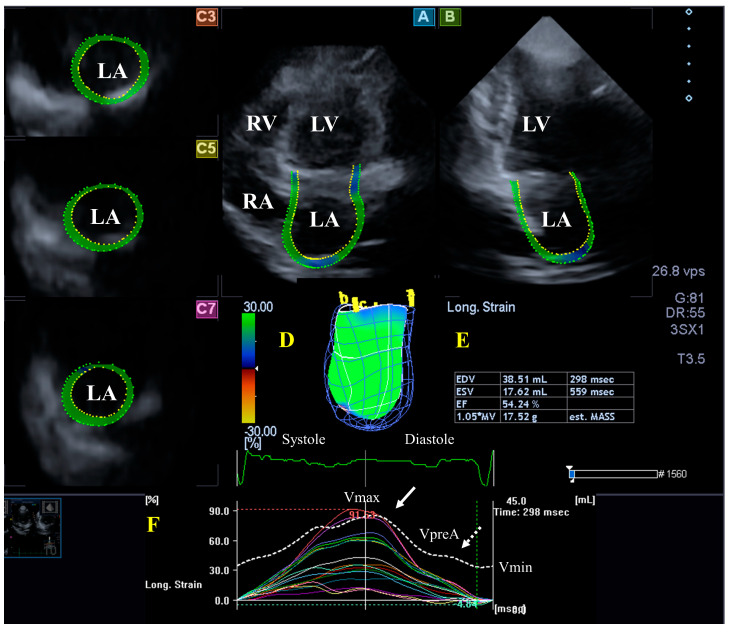
Evaluation of the left atrium (LA) by three-dimensional (3D) speckle-tracking echocardiography. Typically, several views are automatically created during LA assessment using a dedicated vendor-provided software: apical longitudinal four-chamber (**A**) and two-chamber (**B**) views and basal (**C3**), midventricular (**C5**), and superior (**C7**) short-axis views. In a 3D LA model (**D**), the LA volumes (**E**), the LA global (represented by the white curve) and segmental (represented by colored curves) longitudinal (**F**) strain curves with time, and the LA volume changes’ curve with time (dashed white curve) are also presented. Abbreviations: LA = left atrium; LV = left ventricle; EF = ejection fraction; EDV = end-diastolic volume; ESV = end-systolic volume; MASS = LA muscle mass; RA = right atrium; RV = right ventricle; Vmin = minimum LA volume at end-diastole; VpreA = LA volume before atrial contraction at early diastole; and Vmax = maximum LA volume at end-systole.

The LA is on the left posterior side of the heart and is responsible for regulating one-way blood flow between the pulmonary veins and the LV through the MV in normal circumstances. The LA has circumferential (e.g., interatrial band, located at the base) and longitudinal (e.g., septoatrial band, located parietally) muscle bands. The rim of the oval fossa is essential, as the main muscles of the atrium are attached to it. LA function changes with the heart cycle: it acts as reservoir in LV systole, a conduit in early LV diastole, and an active contractile chamber in late LV diastole. The maximum LA volume is detected during end-systole; a pre-atrial contraction volume is seen in early diastole; and the minimal LA volume is measured in late diastole. Emptying fractions and stroke volumes can be calculated from the LA volumes characterizing all phases of LA function. Moreover, LA wall contractility/relaxation during the cardiac cycle could also be characterized by LA strains, similarly to LV [11,32,33]. (Figure 2).

#### 3.2.2. In the Hypereosinophilic Syndrome

According to the guidelines, an enlarged atrium may be observed in HES [26,27]: indeed, 37% of patients show an increased transverse LA dimension [8]. In a small study including seven patients, echocardiography showed atrial enlargement [28]. In the MAGYAR-Path study, elevated LA volumes and (total and active) stroke volumes could be detected in a series of patients with HES, accompanied by decreased peak LA-CS, representing mostly systolic LA reservoir dysfunction, as assessed by 3DSTE [19]. 

### 3.3. Mitral Valve

#### 3.3.1. Under Healthy Circumstances

**Figure 3:**
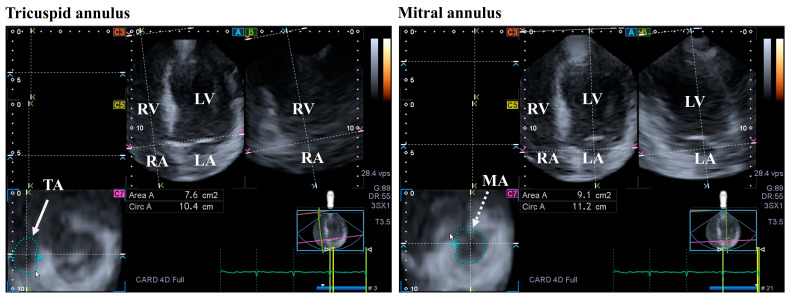
Evaluation of the tricuspid (TA) and the mitral annuli (MA) by three-dimensional (3D) speckle-tracking echocardiography. From the acquired 3D echocardiographic dataset, the following views are created: apical four-chamber (**A**) and two-chamber views (**B**) and a cross-sectional view at the level of the TA/MA optimized in apical four- and two-chamber views (**C7**). The white arrow represents the two-dimensionally projected TA plane, while dashed white arrow represents the two-dimensionally projected MA plane. Abbreviations: RV = right ventricle; RA = right atrium; LV = left ventricle; LA = left atrium; Circ = TA/MA perimeter; and Area = TA/MA area.

The MV is 3D saddle-shaped, with dynamic motion during the cardiac cycle. The MV is made up of the papillary muscles with tendinous chords, the posterior and anterior leaflets, and the fibrous mitral annulus (MA). It opens in the diastole, allowing a unidirectional flow from the LA to the LV, while it closes in the systole. The contraction of adjacent LA and LV myocardial areas considering the heart cycle and the timely occurrence of the contraction of the LA and LV is necessary for the proper contraction of the MA [34,35,36] (Figure 3).

#### 3.3.2. In the Hypereosinophilic Syndrome

According to the guidelines, typical criteria of HES include atrioventricular valve dysfunction due to the fact that the valvular apparatus and the ventricular wall adhered to one another. Restrictive flow pattern across the mitral (or the tricuspid) valve and diffuse thickening of the anterior mitral leaflet are minor criteria that may be observed [26,27]. The atrioventricular valves are almost always affected, showing insufficiency due to restriction of leaflets, mostly affecting the posterior MV leaflet [9]. Echocardiography showed valvular prolapse and insufficiency in a small group of patients with HES [28]. From recent findings presented in the frame of the MAGYAR-Path study, dilation of the MA could be detected in HES cases, accompanied by MA functional deterioration. These abnormalities were found regardless of the presence of hypertension and significant MV regurgitation [20]. 

### 3.4. Aortic Valve

#### 3.4.1. Under Healthy Circumstances

The LV empties through the aortic valve into the aorta, which has three semilunar thin leaflets allowing a one-way blood flow under normal circumstances. The aortic valve opens during systole and closes during diastole [37].

#### 3.4.2. In the Hypereosinophilic Syndrome

No specific aortic valve abnormalities have been demonstrated in a series of patients with HES in the literature.

### 3.5. Aorta

#### 3.5.1. Under Healthy Circumstances

The aorta together with the pulmonary artery are the largest arteries. The aorta forwards the blood from the LV to the systemic circulation. The aorta is not rigid but a flexible tube that helps blood to flow normally with the Windkessel effect. Increased aortic stiffness (reduced distensibility) contributes to LV abnormalities and compromises coronary perfusion [38,39].

#### 3.5.2. In the Hypereosinophilic Syndrome

In a study, the systolic and diastolic diameters of the aorta were similar between matched controls and patients with HES, but increased aortic stiffness was seen in patients with HES [40].

## 4. The Right Heart and the Pulmonary Artery

### 4.1. Right Ventricle

#### 4.1.1. Under Healthy Circumstances

The right ventricle (RV) differs substantially from the LV in shape, structure, and function. The RV is a triangular-shaped heart chamber, when viewed from the side, and crescent-shaped when viewed in a cross-section, increasing in diameter from the apex to the base. The RV wraps around the LV on its right side. The RV fills up from the RA through the TV during diastole, while it empties through the PV into the pulmonary artery during systole. Compared to the LV, the RV muscle is more trabecularized and thinner, resulting in a lower muscle mass. In a normal heart, the contraction of the RV starts with the contraction of the inflow tract and ends with the contraction of the infundibular region. The muscle fibers of the RV can be found deep in the myocardial wall and are responsible for the longitudinal movement of the RV in the basal-to-apical direction, resulting in the shortening of the axis of the RV and in the movement of the TV in the apical direction. The superficially located circumferential muscle fibers of the RV are parallel to the TV fibers and are responsible for the inward movement of the ventricular wall (“bellows” effect). The superficial muscle fibers and, therefore, the movement of the two ventricles are connected. The RV has no rotational mechanics compared to the LV, a structure in which its function has a significant role [41,42,43,44].

#### 4.1.2. In the Hypereosinophilic Syndrome

According to the guidelines regarding the RV, typical criteria for HES include the obliteration of the RV apex, the retraction of the RV apex, and a normal-sized ventricle with enhanced density of the moderator band [26,27]. In HES patients increased RV dimensions were seen, while pericardial effusion was suspected to be present in 32% of the cases [7]. In 10 patients with HES, the RV-EF and RV end-diastolic volumes were normal. In the same study, no patient demonstrated MRI-derived LGE lesions within the free RV wall [30]. 

### 4.2. Right Atrium

#### 4.2.1. In Healthy Subjects

**Figure 4:**
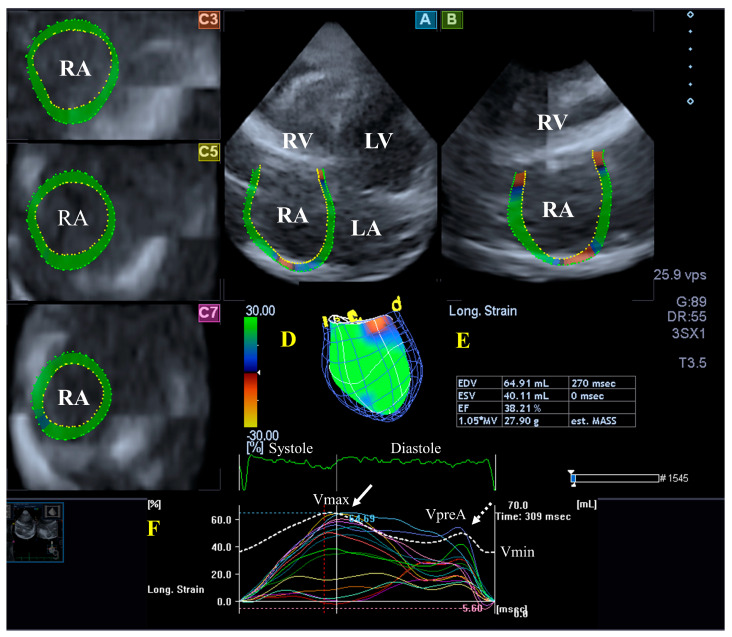
Evaluation of the right atrium (RA) by three-dimensional (3D) speckle-tracking echocardiography. Typically, several views are automatically created during RA assessment using a dedicated vendor-provided software: apical longitudinal four-chamber (**A**) and two-chamber (**B**) views and basal (**C3**), midventricular (**C5**), and superior (**C7**) short-axis views. In a 3D RA model (**D**), RA volumes (**E**), RA global (represented by the white curve) and segmental (represented by colored curves) longitudinal (**F**) strain curves with time, and an RA volume changes’ curve with time (dashed white curve) are also presented. Abbreviations: RA = right atrium; RV = right ventricle; LA = left atrium; LV = left ventricle; EF = ejection fraction; ESV = end-systolic volume; EDV = end-diastolic volume; MASS = LA muscle mass; Vmin = end-diastolic minimum RA volume; VpreA = early diastolic RA volume before atrial contraction; and Vmax = maximum end-systolic RA volume.

Although both atria play an essential role in regulating the one-way flow of blood, there are important differences in their structure. The walls of the RA consist of circumferential and longitudinal muscle bands that are larger than the muscle fibers of the LA. The terminal crest is the most important muscle band arranged longitudinally, while another important muscle band in the RA is formed by the pectinated muscles which are attached to the muscles of the atrioventricular vestibule. The rim of the oval fossa is essential in attaching other muscles, similar to the LA. Regarding its function, the RA has a reservoir phase of its function during systole, with a maximum RA volume during this stage of the cardiac cycle; it serves as a conduit during the early phase of the diastole (pre-atrial contraction RA volume) and acts as a booster pump during late diastole, with a minimal RA volume at that stage. Additional functions of the RA wall are rhythm generation via the sinus node and the secretion of atrial natriuretic peptides, which is modulated by baroreceptors [45] (Figure 4).

#### 4.2.2. In the Hypereosinophilic Syndrome

Limited data are available about RA in HES: for instance, a small study suggested atrial enlargement in HES [28]. In a detailed 3DSTE-based analysis from the MAGYAR-Path study, increased RA volumes with respect to the heart cycle and mild alterations in the RA’s functional properties, including an elevated total and passive atrial stroke volumes representing systolic and early diastolic conduit phase of atrial function, could be demonstrated in series of idiopathic patients with HES. None of the global peak systolic RA strains and RA strains representing a booster pump function at end-diastole showed any abnormalities [21].

### 4.3. Tricuspid Valve

#### 4.3.1. In Healthy Circumstances

The tricuspid valve (TV) is located between the RV and the RA, having a complex structure with a saddle shape. The TV performs dynamic movements throughout the heart cycle: it opens in the diastole and closes in the systole, without regurgitation. Important parts of the TV are the papillary muscles with tendinous chords, septal, anterior, and posterior leaflets, and the fibrous tricuspid annulus (TA), similar to the MV [46] (Figure 3).

#### 4.3.2. In the Hypereosinophilic Syndrome

The TV (together with the MV) is almost always involved in HES, resulting in valvular regurgitation due to leaflet restriction [9]. According to the guidelines, typical features of HES include dysfunction of the atrioventricular valve due to the valvular apparatus’ adhesion to the ventricular wall. A restrictive flow pattern across the TV as a minor criterium can also be seen [26,27]. When HES-related MA and TA abnormalities were compared in the MAGYAR-Path study, although the TA was dilated without functional impairment, the MA abnormalities were more pronounced in HES with vs. without hypertension and significant valvular regurgitation [20]. 

### 4.4. Pulmonary Valve

#### 4.4.1. Under Healthy Circumstances

The pulmonary valve (PV) is located between the pulmonary artery and the RV; its role is to maintain the unidirectional blood flow. Similarly, to the AV, the PV has three semilunar leaflets, opens in systole, and closes in diastole [47].

#### 4.4.2. In the Hypereosinophilic Syndrome

According to the guidelines, diastolic opening of the pulmonary valve may be observed in patients with HES [26,27]. No other PV abnormalities in HES have been suggested in the literature.

### 4.5. Pulmonary Artery

#### 4.5.1. Under Healthy Circumstances

The pulmonary artery is the other largest vessel after the aorta. The pulmonary artery derives from the RV, which is responsible for supplying oxygenated blood to the lungs [44]. 

#### 4.5.2. In the Hypereosinophilic Syndrome

In an early study, three siblings with eosinophilia who developed pulmonary hypertension were reported [48]. Pulmonary hypertension could be detected by echocardiography in patients with HES in another study [28]. 

## 5. Pathophysiologic Background

The etiology of atrial and ventricular remodeling and valvular and vascular abnormalities seen in HES is not known, although there are several potential explanations. In the first acute necrotic phase of HES, myocardial injury occurs due to released cationic toxic proteins from degranulating eosinophils. In the second stage, formation of mural thrombi follows acute myocardial necrosis, which can occur in all regions of the heart cavities and leads to failure of the valves. Thrombosis may be accompanied by a slight increase in the troponin levels, which may be a sign of vascular damage, while the von Willebrand factor, collagen, and the tissue factor may play a role in processes such as fibrin and thrombus formation. Certain factors and cells can trigger procoagulant activity as well. Moreover, disease-associated wall motion hypokinesis may play a role in thrombosis. The last fibrotic phase of the disease eventually leads to restrictive cardiomyopathy and valvular incompetence [7].

These changes may lead to dysfunction of the cardiac chambers and could theoretically explain the findings [49]. Theoretically, the vascular lesions seen in HES may be the direct consequences of the above-mentioned activities as well. However, in accordance with ventricular–arterial coupling, abnormalities in the heart and large vessels can have a mutual effect on each other, explaining the vascular abnormalities in HES [38].

## 6. Clinical Implications

HES is considered to be a rare disorder with heterogeneous origin, and, in the majority of the patients, HES is proved to be idiopathic. Although this has been known for decades and many of the abnormalities described can be confirmed in HES primarily using echocardiography, based on the literature data, these abnormalities can only be seen in a limited number of cases. Moreover, only limited information is available regarding disease-associated abnormalities of the myocardium, valves, and vasculature in series of patients observed via advanced methods, although there has recently been a rapid development in cardiovascular imaging. Hopefully, the information available and detailed above can help clinicians recognize the disease more easily. It would also be important to detect the parameters that are disease-specific and have prognostic power. Moreover, insight into the relationship between the duration of the disease and the identification of instrumental signs of dysfunction in or structure of the heart and large vessels would have clinical importance.

## 7. Conclusions

Summarizing the findings regarding myocardial, valvular, and vascular abnormalities in a series of patients with HES, most studies suggest normal LV volumes with reduced global LV-LS and apical rotation and twist in HES cases, which are accompanied by increased LA volumes and stroke volumes, reduced peak LA-CS (representing systolic abnormalities), and MA dilation and functional deterioration. In the right heart, RV volumes and functional properties have been shown to be preserved, and increased RA volumes, mild functional abnormalities, and a dilated TA without functional impairment have also been seen. Aortic and pulmonary valves have shown no specific disease-related alterations. The vascular abnormalities include increased aortic stiffness without dilation of the aorta and pulmonary hypertension in some cases. These results suggest disease-specific but relatively mild myocardial, valvular, and vascular abnormalities in HES.
